# Current News and Opinion

**Published:** 1888-09

**Authors:** 


### Current News and Opinion.

### FOREIGN CORRESPONDENCE.

*Editor Independent Practitioner:—*

My attention has been called to a letter by Dr. Birdsall, in your issue of May, 1888, in which he anathematizes my contribution on Immediate Root Filling to the last International Medical Congress.

My first objection to the letter of your correspondent is that in his second paragraph he employs quotation marks, even where the form of the sentence does not demand them, evidently with a view of emphasizing the fact that he is quoting my own words; whereas, as a matter of fact, he is only quoting your abstract of the words actually employed. I do not hesitate to say, if you will take the trouble to peruse my original paper, you yourself will admit that it would be difficult to conceive a more misleading and unjust abstract.

I did not preach any doctrines; but merely gave an account of a recorded experience of some five years of immediate root filling, supported by actual statistics, and contrasted these figures with others illustrative of the results of treatment by the dressing method, which seemed to prove to me, as well as others, that far from being "as bad practice as can well be conceived," it is essentially good practice.

With regard to the rest of his letter, it would be only generous to dismiss, without further comment, his absurd, and I must say, amusing conclusions, since they are founded on altogether false premises.

I must, however, say something with regard to the editorial comment appended to that letter. My principal contention is, that you have failed to accord my opinions that respectful treatment at the hands of the reporters to which you rightly admit I am entitled.

The following are the principal errors in the abstract. The statement, that I rarely see my patient again, is a perversion of the statement actually made as to the nature of a University practice. A student's academic career varies from three to five years, sometimes even longer, but it is, of course, evident that if, as sometimes happens, the patient only attends, for dental treatment, near the termination of that career, he disappears in the natural course of events. My practice not being wholly confined to undergraduates, I have an opportunity of seeing the results of my operations in the mouths of Fellows and Professors as well as residents in the town, as they return for the annual or semi-annual inspection. In fact, in the statistical part of my paper, I mention that I had seen 168 cases out of 272, a proportion which I believe would be even greater if the record were made up to the present time.

The practice of cleaning out the pulp-chamber but not the root-canals, and covering up the opening with a disc of paper "moistened with carbolic acid, on one side of which has been taken the twentieth to fifteenth of a grain of arsenic, this side being applied to the fang cavity" is Coleman's method, and not mine, and which I mentioned specially, because the clinical notes of his cases induced me to adopt immediate root-filling some years before it received any prominent attention in America.

In no part of my paper do I mention the combination of creosote with ten or fifteen grains of arsenic.

I never said that most of my cases are dismissed in less than half an hour.

I think, sir, you will admit that such errors as these are quite sufficient to vitiate an abstract scarcely a page in length, and that they constitute anything but respectful treatment.

I must also call your attention to the fact that a full report of my contribution appeared in the January number of the "*Dental Record*" with the illustrative tracings and detailed records of typical cases which unfortunately did not arrive in time for the meeting at Washington.

I am quite willing to stand by the opinions expressed in my contribution, and it seems to me that the only way in which you can do justice to them, after originating such wide-spread misunderstanding, as evidenced by the letter of your correspondent, is by publishing the paper in full.

GEO. CUNNINGHAM, B. A., D. M. D., L. D. S.

We gladly give room for Dr. Cunningham's letter, and would print his paper in full, also, if it had not already been published in the *Record*, and will also shortly appear in the proceedings of the Ninth International Medical Congress. We have, from the first, held opinions similar to those of Dr. C., and believe that we have gone to the extreme in the matter of instrumentation in root-filling —ED.

---

### DRY MOUTH.

At the meeting of the Clinical Society of London, March 17, 1888, Dr. W. B. Haddon read a paper on dry mouth, or suppression of the salivary and buccal secretions. The patient was a woman, sixty-five years old, who had suffered from no affection which could throw light on her present condition. There was no history of family paralysis, or of the prolonged use of belladonna. Her mouth began to get dry some months previous to observation. The tongue was red, devoid of epithelium, cracked in all directions, like crocodile skin, and absolutely dry. The mouth generally was dry, and the mucous membrane smooth, shiny and pale, with a few patches of injection. There was also a deficiency of moisture at the back of the pharynx. The tonsils were natural. The salivary glands, as far as could be made out, were natural in size. Common sensation of the inside of the mouth was unimpaired; but the sense of taste was retarded in consequence of the deficiency of moisture. When the mouth became moist later on, the saliva was found to be slightly acid, and to exert no action on a solution of starch. During this time the mouth had been getting dry, perspiration had notably diminished, and the lachrymal secretion was arrested. The patient received much benefit from the use of jaborandi. A case of similar nature under the care of Mr. Hutchinson was alluded to, and one under the care of Dr. Rowlands, of Liverpool, was communicated by the author of the paper. In conclusion, it was suggested that this condition of dry mouth was due to some disorder of the nervous apparatus.—*Lancet*, March, 17, 1888.

### CANADOL AS A LOCAL ANÆSTHETIC.

Canadol is one of the constituents of a heavy naphtha-ether (No. II.) obtained by the fractional distillation of American naphtha. It represents a limpid, very volatile, easily inflammable fluid, possessing a strongly pronounced benzine odor. The liquid is insoluble either in water or in alcohol. According to a report of Dr. J. P. Plüshkoff, of Professor Hadensky's clinic, canadol was atomized by means of Richardson's apparatus, the nozzle of the latter being kept at a distance of five or six centimetres from the operation field. The results were most satisfactory. The integuments became frozen in the interval, varying from thirty to ninety seconds; in a majority of the cases complete anæsthesia was obtained in less than a minute Only one of the patients complained of pain when some deep lymphatic glands were incised. In the remaining cases the operation was quite painless (as well as bloodless) from the beginning to the end. The healing process did not differ from the normal in any of the cases.—*Ther. Gazette.*

### OIL OF PEPPERMINT AS AN ANTISEPTIC.

W. L. Braddon has instituted extensive experiments to discover, if possible, an efficient microbicide which would be sufficiently harmless to human beings to answer for internal use. In a communication to the *Lancet* the author reports that these experiments were carried out, first under conditions as nearly as possible identical with those which obtain with wounds, etc., the relative powers of carbolic acid, iodine, iodoform, corrosive sublimate, and peppermint being compared. The observer considers the complete superiority of the last completely proved, and has tried its powers in actual practice with most excellent results.—*N. Y. Med. Times.*

It has long been known in dental surgery that the essential oils are good antiseptics. Our object in quoting the above is to show that the medical profession is also awakening to a knowledge of the fact.

### REIMPLANTATION OF A TREPHINE BUTTON OF BONE.

Herbert L. Burrell, M. D., Boston, Mass., reports a case of exploratory trephining in a boy, æt. 13, in which the button was placed in an antiseptic solution during the examination. It was then replaced and the periosteal flap and scalp sutured over it, the wound completely uniting in two weeks. The child dying eight months later, an opportunity was offered for examining the skull. The trephine button was found united by bony union throughout. The author suggests that this opens an important field for exploratory action, since the cranial opening can be cured even more readily than an abdominal incision.—*Boston Med. and Surg. Jour.*, March 29, 1888.

---

THE following is taken from the *Hartford* (Conn.) *Times:*

"One Dollar.—All ordinary gold stoppings at the extremely low price (for first-class work) of one dollar. Our offer for sixty days ended on May 1st, but we have decided to continue it until further notice. All ordinary gold stoppings at the extremely low price of one dollar for first-class work. All other branches at proportionate prices. Dr. G. A. Mills, Oral Surgeon and Dentist, No. 18 Garden street, four doors from Asylum avenue, two blocks west of the N. Y., N. H. & H. R. R. Depot"

Advertisements in the public press are public property, and by quoting them it is presumable that we are only doing the advertiser a favor. The above party being a contributor to one of our esteemed contemporary journals, we have been requested by a dentist in practice to publish his full card, for which we make no extra charge. Comment is unnecessary.—[ED.

---

THE officers elected for the ensuing year in the Pennsylvania State Society were:

*President*—H. C. Register.

*First Vice-President*—J. C. M. Hamilton.

*Second Vice-President*—W. H. Fundenberg.

*Recording Secretary*—J. R. C. Ward.

*Assistant Secretary*—C. V. Kratzer.

*Corresponding Secretary*—P. K. Filbert.

*Treasurer*—L. Ashley Faught.

*Board of Censors*—S. H. Guilford, G. L. Robb, J. S. Goshhorn, W. H. Trueman, and Alonzo Boice.

*Board of Examiners*—E. C. Kirk, W. E. Van Arsdel, and C. S. Beck.

The session adjourned to meet at Cresson Springs on the last Tuesday in July, 1889.

---

LAPLACE'S sublimate solution is more antiseptic and less irritating than ordinary solutions. For irrigation—sublimate 1, acid tartaric 5, dist. water 1000. Gauze and cotton for dressings are soaked for two hours in—sublimate 5, acid tartaric 20, dist. water 1000. We should prefer solutions of half the strength.

---

THE next regular meeting of the Illinois State Board of Dental Examiners will be held at 10 A. M., on Monday, Sept. 17th, in the State House, at Springfield. Candidates for examination must report at that time.

CHARLES E. KOCH, Sec. Ill. State Board.

---

IODOFORM, while not a germicide as such, becomes so by evolving free iodine. Laboratory evidence is not sufficient to make us abandon an agent which, used with discretion, is of immense value, especially in the surgery of the cavities.

